# Heme cross-feeding can augment *Staphylococcus aureus* and *Enterococcus faecalis* dual species biofilms

**DOI:** 10.1038/s41396-022-01248-1

**Published:** 2022-05-19

**Authors:** Jun-Hong Ch’ng, Mugil Muthu, Kelvin K. L. Chong, Jun Jie Wong, Casandra A. Z. Tan, Zachary J. S. Koh, Daniel Lopez, Artur Matysik, Zeus J. Nair, Timothy Barkham, Yulan Wang, Kimberly A. Kline

**Affiliations:** 1 Department of Microbiology and Immunology, National University of Singapore, Singapore, Singapore; 2 Department of Surgery Yong Loo Lin School of Medicine, National University of Singapore, Singapore, Singapore; 3 Infectious Disease Translational Research Program, National University Health System, Singapore, Singapore; 4 Singapore Centre for Environmental Life Sciences Engineering, National University of Singapore, Singapore, Singapore; 5 Singapore Centre for Environmental Life Sciences Engineering, Nanyang Technological University, Singapore, Singapore; 6 Nanyang Technological University Institute for Health Technologies, Interdisciplinary Graduate School, Nanyang Technological University, Singapore, Singapore; 7 Singapore Centre for Environmental Life Sciences Engineering, Interdisciplinary Graduate Program, Nanyang Technological University, Singapore, Singapore; 8 Department of Laboratory Medicine, Tan Tock Seng Hospital, Singapore, Singapore; 9 Singapore Phenome Center, Lee Kong Chian School of Medicine, Nanyang Technological University, Nanyang, Singapore; 10 School of Biological Sciences, Nanyang Technological University, Singapore, Singapore

**Keywords:** Biofilms, Clinical microbiology, Bacteriology, Microbial ecology

## Abstract

The contribution of biofilms to virulence and as a barrier to treatment is well-established for *Staphylococcus aureus* and *Enterococcus faecalis*, both nosocomial pathogens frequently isolated from biofilm-associated infections. Despite frequent co-isolation, their interactions in biofilms have not been well-characterized. We report that in combination, these two species can give rise to augmented biofilms biomass that is dependent on the activation of *E. faecalis* aerobic respiration. In *E. faecalis*, respiration requires both exogenous heme to activate the *cydAB*-encoded heme-dependent cytochrome *bd*, and the availability of O_2_. We determined that the ABC transporter encoded by *cydDC* contributes to heme import. In dual species biofilms, *S. aureus* provides the heme to activate *E. faecalis* respiration. *S. aureus* mutants deficient in heme biosynthesis were unable to augment biofilms whereas heme alone is sufficient to augment *E. faecalis* mono-species biofilms. Our results demonstrate that *S. aureus*-derived heme, likely in the form of released hemoproteins, promotes *E. faecalis* biofilm formation, and that *E. faecalis* gelatinase activity facilitates heme extraction from hemoproteins. This interspecies interaction and metabolic cross-feeding may explain the frequent co-occurrence of these microbes in biofilm-associated infections.

## Introduction

Biofilms consist of a sessile community of microbes embedded within a matrix of extracellular polymeric substances. Biofilms represent the dominant mode of bacterial life, and biofilm cells exhibit different patterns of behaviour compared to planktonic cells [1]. Characteristically, biofilms confer protection to chemical and physiological stresses [2], sheer forces [3, 4], predation [5], and to antibiotic-mediated clearance [6]––rendering biofilm-associated infections difficult to treat. Behaviourally, biofilm-embedded cells are extremely heterogeneous, exhibiting varying levels of metabolic activity (from very active to quiescent) [7, 8], transcriptional profiles (depending on the microenvironment) [9, 10] and higher frequencies of genetic exchange (via transformation, conjugation and transduction) [11, 12].

Biofilms are frequently polymicrobial, comprising of different species, phyla or kingdoms interacting within the complex community [13]. Typically categorized as commensal, antagonistic or synergistic for simplicity, these multi-species interactions are multifaceted and evolve temporally with fluctuations in the microenvironment such as pH, temperature, oxygen, nutrient and waste levels, and quorum-sensing signals [14]. Although in-depth characterization of molecular interactions in polymicrobial biofilms is limited, controlled studies have enabled the identification of critical mediating compounds important for interspecies interactions. Notable mediators include c-di-GMP [15, 16], AI-2 [17, 18], alarmone ppGpp [19, 20], bacteriocins [21–23], siderophores [24], L-ornithine [25], lactic acid [26], lipoteichoic acid [27, 28], glycans [29] and indole [30, 31], and these have been reviewed elsewhere [14, 32–42].

*E. faecalis* and *S. aureus* are both opportunistic pathogens and are among the leading causes of nosocomial infections [43, 44]. The biofilm-forming potential of each species is well documented [33, 45] and both have been implicated in biofilm-associated infections such as endocarditis [46, 47], urinary tract infections [48, 49] and chronic wounds [50, 51]. Although, *E. faecalis* and *S. aureus* are commonly co-isolated in chronic wounds such as diabetic foot ulcers [52], venous leg ulcers [53] and pressure wounds [54], studies of their interactions are largely limited to the transfer of vancomycin resistance genes from *E. faecalis* to *S. aureus* in clinical settings [55–57]. Therefore, in this study, we explored the molecular interactions between *E. faecalis* and *S. aureus* in biofilms.

Aerobic respiration in *E. faecalis* requires exogenous heme as a cofactor for cytochrome *bd* [58, 59]. Enterococci are unable to synthesize heme due to an absent TCA cycle that prevents the formation of porphyrin precursors. Through a yet unknown importer, heme is taken into the cell and incorporated into cytochrome *bd* (CydAB), which then converts terminal demethylquinol (DMKH_2_) to demethylmenaquinone (DMK), consuming O_2_ and releasing H_2_O in the process [60]. DMK is reduced by NADH:quinone oxidoreductase back into DMKH_2_ consuming NADH. Importantly, Enterococci do not express other membrane-embedded electron carriers like ubiquinone or menaquinone [60], but only make DMK which is a modified menaquinone lacking a 2-methyl group [61]. Cytochrome *bd* is the key respiratory enzyme for *E. faecalis* and contains two subunits (CydA and CydB) with three cytochromes, *b*_*558*_, *b*_*595*_ and *d*. The translocation of a proton by cytochrome *bd* establishes a proton motive force that, when coupled with F0F1-ATP synthase, generates ATP [62, 63]. Additionally, *cydC* and *cydD* are necessary for cytochrome *bd* production and have been suggested to be involved in heme transport and/or cytochrome *bd* assembly [60]. Interestingly, reduction of O_2_ by DMK induces extracellular superoxide production, the latter of which was inhibited by exogenous heme [64], while a functional electron transport chain sensitizes *E. faecalis* to oxidative burst and decreased its survival in human blood [65].

In this study, we show that *S. aureus*-derived heme is required to activate *E. faecalis* aerobic respiration, leading to augmented *E. faecalis* growth and augmented dual-species biofilm production. We speculate that this interspecies cross-feeding of heme, where one species consumes metabolic end-products from another, may affect mixed species infection outcomes in heme-restricted host and environmental niches.

## Materials and methods

### Bacterial strains and growth conditions

Strain, isolate and transposon library details are available in Supplementary Information. Overnight cultures of *E. faecalis* were grown in Brain Heart Infusion broth (Becton-Dickinson, United States) whereas *S. aureus* grown in Tryptone Soy Broth (Oxoid, England). Agar Technical Powder No. 3 (Oxoid, England) was used for agar plates. Strains were cultured under static conditions at 37 °C. Overnight cultures were spun down and washed once in phosphate-buffered saline prior to normalization. An OD_600_ of 1.3 and 3.0 for *E. faecalis* and *S. aureus* respectively gave about 1 × 10^9^ CFU/ml. MRSA Select II agar (Bio-Rad Laboratories, United States) was used to select *S. aureus* USA300LAC from mixed-species cultures whereas rifampicin (25 µg/ml) was added to BHI agar to select for OG1RF. Rifampicin (Sigma-Aldrich, United States) was dissolved in methanol to make a stock of 25 mg/ml and stored at −20 °C. Hemin (Sigma-Aldrich, United States) was dissolved in DMSO to make a stock of 25 mg/ml whereas human hemoglobin (Sigma-Aldrich) was dissolved in dH_2_0 and filter sterilized to make stock of 10 mg/ml. For hemin and hemoglobin supplementation, a final concentration of 25 μg/ml and 10 μg/ml respectively were used unless otherwise stated.

### Biofilm assays

Normalized cultures of *E. faecalis* and *S. aureus* were mixed (with equal CFU) in the ratio of 1:1 for dual-species biofilms and 8 µl of single- or dual-species cultures were inoculated in 200 µl of Tryptone Soy Broth in a 96-well flat transparent plates (Thermo Fisher Scientific, United States) and incubated under static conditions at 37 °C for five days unless otherwise stated. For anoxic experiments, plates were instead incubated in an anaerobic jar (Merck, Singapore) with a gas pack (Becton Dickinson, United States) and incubated at 37 °C for five days. Details on Crystal Violet staining and CFU determination is available in Supplementary Information. For biofilm oxygen consumption rate (OCR) assays, 5-day biofilms were grown directly in Seahorse XFe96 FluxPak 96-well plates, with 80 µl of inoculated media added per well. Planktonic cells were removed with three washes of 80 µl PBS using a 96-channel pipettor to prevent cross-contamination and the remaining biofilms were resuspended in 80 µl of PBS. After three baseline measurements were taken, 30 µl of fresh TSB was injected into the wells and OCR measured for 1 h using standard parameters.

### Growth kinetics

Normalized cells were diluted 100× in PBS and 8 µl of diluted cells were added to 200 µl TSB in a 96-well plate. The plate was incubated in Tecan Infinite M200 PRO Spectrophotometer at 37 °C for 20 h and absorbance (600 nm) recorded for every 15 min after shaking the plate for 3 s.

### Transposon library screen and transposon mutants

The *E. faecalis* Transposon Library was provided by Gary M. Dunny [66] and *S. aureus* transposon mutants were from Nebraska Transposon Library [67] provided by the Network on Antimicrobial Resistance in *Staphylococcus aureus* (NARSA) and distributed by BEI resources, NIAID. Details on the screening of the *E. faecalis* transposon library [66, 68] are available in Supplementary Information. The primary screen of dual-species biofilms was performed by adding 5 µl of transposon mutants grown overnight and 3 µl of USA300LAC overnight cultures to 200 µl TSB in the 96-well plate. Each plate had controls of parental strains of OG1RF + USA300LAC. Plates were incubated at 37 °C for five days in a humidified incubator and biofilms quantified by crystal violet. *E. faecalis* transposon mutants that showed reduced staining were selected as hits. Confirmation of primary screen hits was done via a secondary screen (three biological replicates) and secondary screen hits that consistently showed reduced dual-species biofilms were subject to further validation. Transposon mutants without defects in growth kinetics and single-species biofilms, but with reduced dual-species biofilms, were shortlisted as validated hits.

### LC–MS for heme quantification from cell pellet

*E. faecalis* strains (OG1RF, *cydA::Tn*, *cydD::Tn* and *cydC::Tn*) were grown overnight in 20 ml of TSB with or without supplementation of 5 µg/ml hemin. Cell pellets were washed twice with PBS before being normalized by OD600. Cell pellets were mixed with 100 µl of lysozyme (10 mg/ml), incubated in a water bath at 37 °C for 1 h and then stored at −80 °C until LC–MS quantification.

*S. aureus* strains (USA300LAC, *hemA::Tn*, *hemL::Tn*, *hemB::Tn* and *hemE::Tn*) were grown overnight in 20 ml of TSB were resuspended in 950 µl of P1 Buffer. 50 µl of lysostaphin (5 mg/ml) was added and incubated at 37 °C for 1 hr and then stored at −80 °C until LC–MS quantification.

LC–MS details are available in Supplementary Information.

### Gelatinase activity assay

Gelatin tubes were made by combining 3 g of gelatin and 3.7 g of BHI powder with 100 ml water that was autoclaved before 3 ml was added into 15 ml tubes. *E. faecalis* colonies stabbed into the gelatin tubes prior to overnight incubation at 37 °C. Tubes were then refrigerated at 4 °C for an additional 30 min before the tubes were gently tilted to observe for liquification of gelatin due to gelatinase activity [69]. Any liquification was indicative of positive gelatinase activity. Parental strain OG1RF and OG1RFΔ*gelE* served as positive and negative controls respectively, and assays were repeated four times to confirm gelatinase activity.

### Statistics

Statistical analyses used GraphPad Prism 7 software (version 7.03) (GraphPad, United States). The data was analyzed through unpaired students T-test or one-way analysis of variance (ANOVA) with Tukey’s post-hoc testing for multiple inter-group comparison, or with Dunnett’s post-hoc testing if comparing to a specific control group. Significance for *p* < 0.05 are reported.

### Ethics

All procedures were performed in accordance with Nanyang Technological University Research Integrity Policy. Anonymized and pure bacterial strains used in this study did not require IRB approval.

## Results

### *S. aureus* augments *E. faecalis* growth within biofilms and overall biomass accumulation

**Fig. 1:**
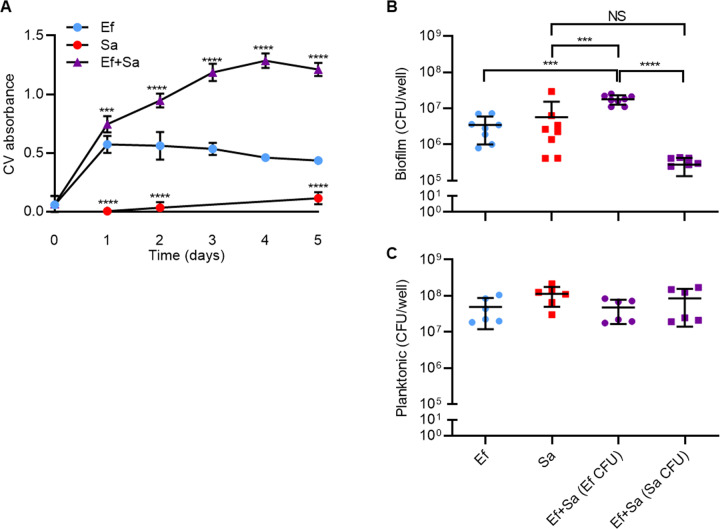
Single- and dual-species in vitro biofilms. **A** *E. faecalis* (Ef) and *S. aureus* (Sa) biofilms were grown in 96-well plates alone or in combination (1:1) over five days before biofilm was quantified by crystal violet (CV). Day 0 refers to 1 h post-inoculation. Data shows mean and SD (*N* ≥ 3) with ***p < 0.001 and ****p < 0.0001 when compared to *E. faecalis*-only biofilms of the same time point using 1-way ANOVA with Tukey’s post-hoc test. **B** Day 5 biofilm and (**C**) planktonic cells of Ef or Sa or both were collected and CFU/well determined using selective agar. Data shows mean and SD (*N* ≥ 6) with ***p < 0.001 and **** *p* < 0.0001 using 1-way ANOVA and Tukey’s post-hoc test.

Since *S. aureus* and *E. faecalis* are frequently co-isolated during biofilm-associated infections [55], we first examined the consequence of dual species growth on biofilm biomass accumulation. Using a modified polystyrene microtiter assay followed by crystal violet (CV) staining [70], we observed that *E. faecalis* strain OG1RF (a rifampicin and fusidic acid resistant derivative of a human oral isolate OG1, commonly used in molecular manipulation and virulence studies and used throughout this study) biofilm biomass peaked at day 1 and plateaued for the remaining four days, whereas *S. aureus* (strain USA300LAC) formed very poor biofilms under these experimental conditions (Fig. 1A). By contrast, following inoculation of *E. faecalis* together with *S. aureus* at a ratio of 1:1, dual-species biofilms showed significantly greater biomass from day 1 and peaked at day 4. Since the greatest difference in dual-species biomass accumulation occurred at day 4 and 5, we chose day 5 for subsequent assays.

To determine if increased bacterial growth within dual species biofilm contributed to the augmented biomass, biofilms were manually disrupted and CFU determined for both single and dual species biofilms. In day 5 biofilms, we observed a statistically significant 5-fold increase in *E. faecalis* CFU within dual-species biofilms compared to mono-species biofilms (Fig. 1B) which correlated with the increased biomass observed by crystal violet staining. *E. faecalis* out-numbered *S. aureus* by approximately 60-fold in dual-species biofilms. Surprisingly, although *S. aureus* produced very little biofilm biomass compared to *E. faecalis* (Fig. 1A), CFU equivalent to that of *E. faecalis* were recovered, suggesting that *E. faecalis* may produce more abundant biofilm matrix than *S. aureus*. Compared to the increased *E. faecalis* biomass and CFU in the presence of *S. aureus*, there were no significant differences in CFU sampled from non-adherent (planktonic) volume of the same biofilm wells, suggesting that *E. faecalis* growth augmentation by *S. aureus* is specific to biofilms (Fig. 1C).

### Dual-species biofilm augmentation is strain dependent

**Fig. 2:**
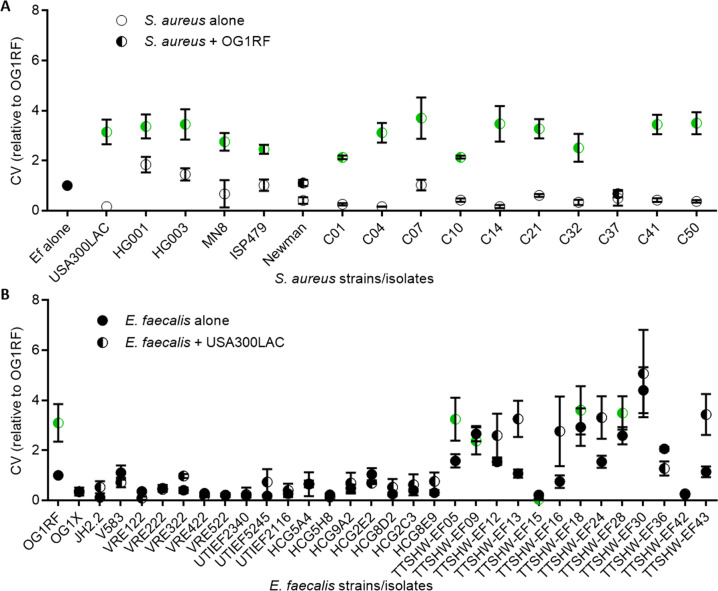
Augmentation of biofilms with multiple *E. faecalis* and *S. aureus* laboratory strains and clinical isolates. **A** Six *S. aureus* laboratory strains (USA300LAC–ISP479) and ten patient isolates (C01–C50) were grown alone or with *E. faecalis* (OG1RF) for five days before biofilms were quantified by crystal violet (CV). Results show biofilm levels normalized to OG1RF-only control, with mean and SD displayed (*N* ≥ 3). Points colored green are significantly different to respective single species biofilms by *p* < 0.05 using 1-way ANOVA with Bonferroni’s post-hoc test (details in Supplementary Table 1). **B** Four laboratory strains of *E. faecalis* (OG1RF-V583), together with 28 *E. faecalis* patient isolates (VRE122-TTSHW-EF43) were grown for five days alone or with *S. aureus* (USA300LAC) before biofilm was quantified by CV. Results show biofilm levels normalized to OG1RF-only controls, with mean and SD (*N* ≥ 3). Points colored green are significantly different to respective single species biofilms by *p* < 0.05 using 1-way ANOVA with Bonferroni’s post-hoc test (details in Supplementary Table 2).

To investigate whether *S. aureus* augmentation of *E. faecalis* biofilm is a phenomenon unique to the strains used in the initial studies (*E. faecalis* strain OG1RF and *S. aureus* strain USA300LAC), we assayed for biofilm augmentation using five additional commonly used *S. aureus* laboratory strains, as well as ten clinical wound isolates. We observed that 14 out of the 16 tested *S. aureus* strains/isolates showed a significant biofilm biomass increase when co-cultured with *E. faecalis* OG1RF (Fig. 2A and Supplementary Table 1). Newman strain and isolate C37 did not augment mixed species biofilm biomass, potentially because of anti-enterococcal activity or biofilm-restricting properties of these strains that are discussed later. Together, the data indicates that *S. aureus* strain variation influences the nature of the dual-species relationship with *E. faecalis* vis-à-vis biofilm development.

We next investigated if the *E. faecalis* strain variation also affects dual-species biofilm augmentation by testing three additional *E. faecalis* laboratory strains and 28 clinical isolates derived from bloodstream (VRE isolates), wound (TTSHW-EF05 to EF43), urinary tract infections (UTIEF isolates), or the healthy gastrointestinal tract of children (HCG isolates). In contrast to the relative consistent ability of *S. aureus* strains to augment dual-species biofilms, augmentation observed during co-culture with *S. aureus* USA300LAC was much more heterogenous with only six out of 32 strains/isolates showing augmented biofilms (Fig. 2B and Supplementary Table 2). Of these six strains, the degree of augmentation observed varied between 2.06–3.10 fold (Supplementary Table 2). Of the 26 that did not produce augmented biofilms, 17 showed single-species biofilm levels that were lower than OG1RF under the conditions used (defined arbitrarily as <0.5× the biofilm levels of OG1RF), five were defined as having high biofilm levels (>2× the biofilm levels of OG1RF), and four had similar levels of biofilm to OG1RF (>0.5× but <2× of OG1RF) (Supplementary Table 2). Taken together, we conclude that many *E. faecalis* isolates are not susceptible to biofilm augmentation by USA300LAC. This is in keeping with other studies showing strain differences (not just species composition) profoundly affects the nature of microbial interactions [71–73].

### *E. faecalis* transposon screen identifies *menA* and *cydA* to be crucial for dual-species biofilm augmentation

**Fig. 3:**
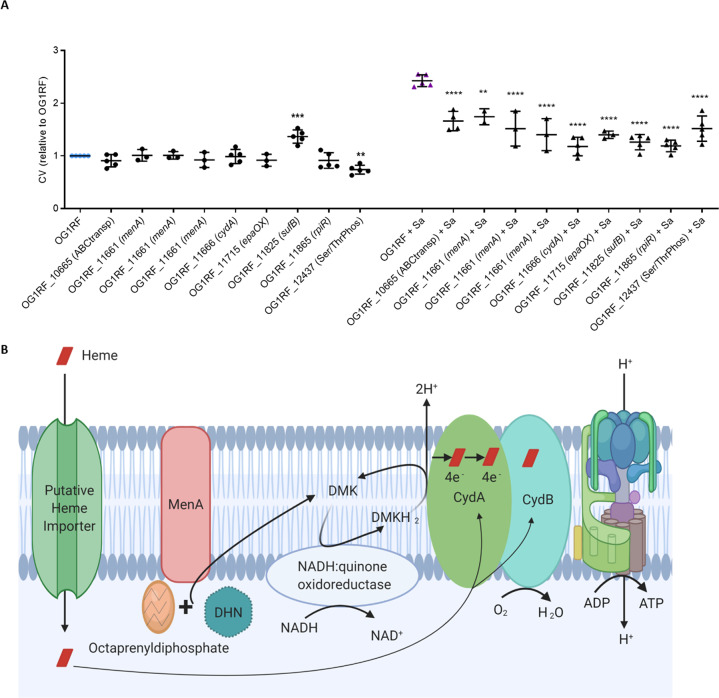
Validation of *E. faecalis* transposon library screen hits. **A** The parental strain (OG1RF) and nine transposon mutants were grown alone (circles), or together with *S. aureus* (Sa, USA300LAC, triangles) for five days before biofilm levels were determined by crystal violet (CV). Data shows mean and SD relative to OG1RF-only control (*N* ≥ 3). **p < 0.01, ***p < 0.001 and ****p < 0.0001 when compared to respective OG1RF-only or OG1RF + Sa control biofilms using 1-way ANOVA with Dunnett’s post-hoc test. **B** Oxidative respiration of *E. faecalis* requires MenA, CydA, CydB, heme and O_2_.

To determine the mechanism by which *E. faecalis* CFU are increased in the presence of *S. aureus*, leading to dual-species biofilm augmentation, we screened a near-saturated *E. faecalis* transposon mutant library [66] for mutants that displayed altered dual-species biofilm accumulation, using the same microtiter CV biofilm assay. After secondary screening to eliminate any mutants that were attenuated in biofilm formation, we validated nine mutants in seven unique genes that reduced dual-species biofilms (Fig. 3A).

Amongst these was *epaOX* that encodes a glycosyltransferase involved in the production of cell wall rhamnopolysaccharide enterococcal polysaccharide antigen (Epa), is responsible for *E. faecalis* biofilm structure and stability in response to antibiotic stress [74], and is a determinant of biofilm-associated antibiotic resistance [75]. Both the transposon mutant and a clean deletion mutant of *epaOX* showed a reduction in dual-species biofilm levels relative to the single species control which was restored to parental strain levels in the plasmid-complemented strain (Supplementary Fig. 1a) after correcting for variations in single species biofilm levels (Supplementary Fig. 1b). As such, it seems likely that *epaOX* is involved in augmented dual-species biofilms through EPS production.

The roles of four additional validated gene products identified in the transposon screen (an ABC transporter, SufB, RpiR, and a Ser/Thr Phosphatase, Fig. 3A) were not pursued. The essentiality of *sufB* had been suggested in some studies [76] but deemed otherwise by others [58], and its involvement in respiration, via FeS cluster assembly, merits future attention. Most importantly, we identified three unique *menA* (OG1RF_11661) and one *cydA* (OG1RF_11666) transposon mutants that failed to undergo dual species augmentation. Both genes encode proteins participating in oxidative respiration (Fig. 3B) [77] prompting closer investigation into this process.

### *E. faecalis* OG1RF biofilm augmentation by *S. aureus*, heme and hemoglobin requires O_2_ and *cydABCD*

**Fig. 4:**
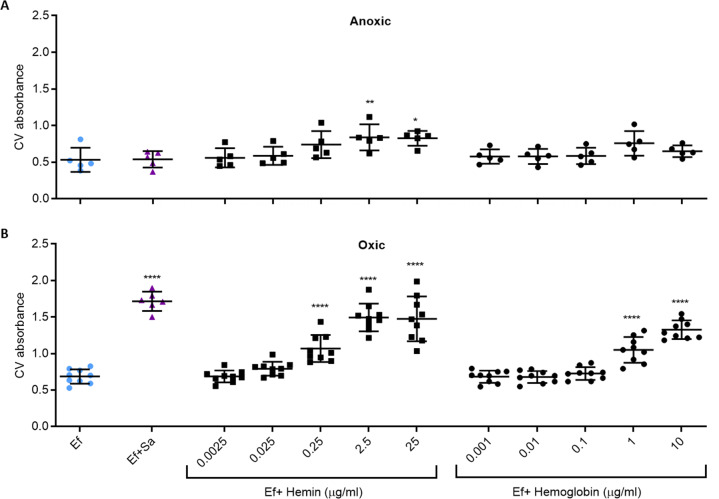
E. faecalis biofilms augmented under oxic and anoxic conditions. E. faecalis (Ef) biofilms were grown for five days under **A** anoxic and (**B**) oxic conditions, alone or in the presence of S. aureus (Sa), hemin or hemoglobin (Hb) before biofilm was quantified by crystal violet (CV). Data shows mean and SD of CV absorbance. ****p < 0.0001 when compared to Ef-alone control using Dunnett’s post-hoc test (*N* ≥ 4).

We hypothesized that *E. faecalis* respiration is necessary for dual-species biofilm augmentation, and to test this, biofilms were cultured in oxic or anoxic conditions. As predicted, dual-species biofilm augmentation was not observed in anoxic conditions (Fig. 4A) but was increased by over 2-fold in oxic conditions (Fig. 4B), demonstrating that oxygen is required for dual-species biofilm augmentation.

Since the utilization of oxygen by *E. faecalis* is dependent cytochrome *bd* which requires the incorporation of exogenous heme as a cofactor (*E. faecalis* does not synthesize heme [63, 78, 79]), we directly investigated the effect of free and conjugated heme (hemin and haemoglobin respectively) on *E. faecalis* biofilms. Supplementation with either source significantly increased biofilm biomass by over two-fold in oxic conditions (Fig. 4B) and was accompanied by an increase in oxygen consumption rate (Supplementary Fig. 2). Additionally, *E. faecalis* biofilm growth kinetics with hemin supplementation were measured, with a rapid increase in biofilm staining observed to plateau after day 1 (Supplementary Fig. 3). Under anoxic conditions, hemin supplementation minimally impacted biofilm levels and hemoglobin had no effect (Fig. 4A).

**Fig. 5:**
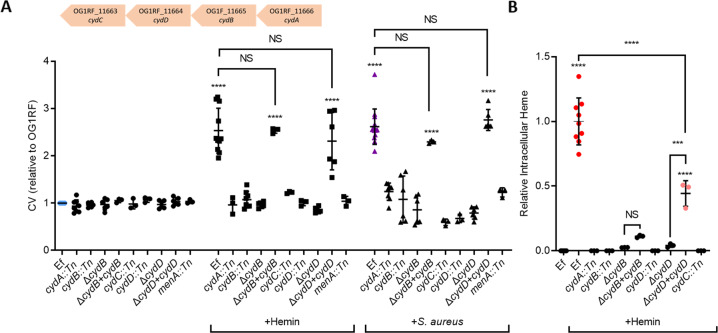
The *E. faecalis cydABCD* operon is required for biofilm augmentation by hemin and *S. aureus*, and for heme uptake. **A** Transposon mutants for *cydA*, *cydB*, *cydC*, *cydD* and *menA*, deletion mutants of *cydB* and *cydD*, their chromosomally complemented strains, along with parental wild-type OG1RF, were grown alone, in the presence of hemin (25 μg/ml), or with *S. aureus* (Sa, USA300LAC) for five days before biofilm was quantified with crystal violet (CV). Insert shows *cydABDC* operon and arrows indicate the direction of transcription. **B** Transposon and deletion mutants for *cydA*, *cydB*, *cydC* and *cydD*, along with complementary strains and parental wild-type OG1RF, were grown overnight in the presence of hemin (5 μg/ml) before cells were pelleted, lysed and analyzed by LC–MS for intracellular heme. Data shows mean values and SD. *p < 0.05, **p < 0.01, ***p < 0.001 and ****p < 0.0001 when compared to respective OG1RF control (first of each group) using Tukey’s post-hoc tests (*N* ≥ 3).

Cytochrome *bd* complex consists of two subunits that are encoded by *cydA* and *cydB*. The same operon (Fig. 5A insert) includes *cydC* and *cydD*, which encode an ATP-binding cassette (ABC)-type transporter required for the expression of a functional cytochrome *bd* complex [63, 80]. As a functional *cydABCD* operon was presumed to be required for aerobic respiration to occur, we tested if disruptions of *cydB*, *cydC* and *cydD* would also attenuate the *E. faecalis* biofilm response to both *S. aureus* and hemin. Biofilm augmentation by both hemin and *S. aureus* was significantly impaired in all the tested transposon mutants of the four genes (Fig. 5A), indicating that that a functional operon was required for augmentation. Deletion mutants of *cydB* and *cydD* likewise failed to augment in the presence of hemin and *S. aureus*, with augmentation completely restored in the complementary strains (Fig. 5A). Additionally, the absence of heme- or hemoglobin-induced augmentation in the *menA* transposon mutant shows the requirement for demethylmenaquinone for oxidative respiration to occur (Figs. 5A and 3B).

To ensure that the absence of *E. faecalis* biofilm augmentation by *S. aureus* in the *cydABDC* transposon and deletion mutants was not due to their out-competition by *S. aureus*, the planktonic cells in single- and co-cultured wells were enumerated on selective agar after one day. Of the tested strains, only *epaOX::Tn* had lower CFUs compared to the OG1RF parental strain, both when grown alone and in the presence of *S. aureus*, allowing us to exclude out-competition by *S. aureus* as a confounding factor (Supplementary Fig. 4).

To differentiate the involvement of cytochrome *bd* (CydAB) from the heterodimeric ABC transporter (CydDC) in *E. faecalis* biofilm augmentation, intracellular heme was measured in the cell pellets of the respective transposon and deletion mutants and complementary strains by mass spectrometry. Consistent with the inability of *E. faecalis* to synthesize heme, heme was only detected when *E. faecalis* was cultured in TSB supplemented with hemin, but not in the unsupplemented TSB control (Fig. 5B). Intracellular heme was negligible or not detected in any of the *cyd* mutants when grown in hemin-supplemented TSB (Fig. 5B), suggesting that this operon is essential for heme import. Complementation of *cydD*, but not of *cydB*, partially restored intracellular heme levels (Fig. 5B), potentially due to transcriptional differences arising due to *trans* chromosomal complementation with a non-native (*srtA*) promoter. Notwithstanding, partial restoration of intracellular heme levels in the *cydD* complement indicates that the functional product of the *cydDC* operon is required for heme import. Taken together, these data support that aerobic respiration in *E. faecalis* is activated by heme, hemoglobin or *S. aureus*, leading to augmented biofilm formation, and that import of heme into *E. faecalis* is mediated by CydDC.

### Heme biosynthesis in *S. aureus* is responsible for dual-species biofilm augmentation

Since *E. faecalis* cannot synthesize heme [63, 79], we hypothesized that *S. aureus*-derived heme might enable the activation of cytochrome *bd*, giving rise to augmented dual-species biofilms. If so, *S. aureus* mutants deficient in heme biosynthesis should be unable to augment *E. faecalis* biofilms. We identified *S. aureus* transposon mutants in four key heme biosynthesis enzymes (*hemA, hemB, hemE* and *hemL*) in the *S. aureus* Nebraska transposon mutant library [67]. To verify the loss of heme biosynthetic activity, we performed growth assays to query the expected growth defect when exogenous heme was limited [81] and quantified heme in cell pellets by LC–MS. Only *hemB::Tn* displayed a growth defect (Supplementary Fig. 5a) that was restored upon heme supplementation (Supplementary Fig. 5b). The *hemB* mutant was also the only one that showed a 40-fold reduction in heme within cell pellets (Supplementary Fig. 5c). These data indicated that the *hemB* transposon mutant was the only bona fide heme-defective mutant of the four. The *hemA*, *hemE* and *hemL* mutants may have acquired secondary mutations, a documented caveat to this library [82], enabling them to bypass these mutations.

**Fig. 6:**
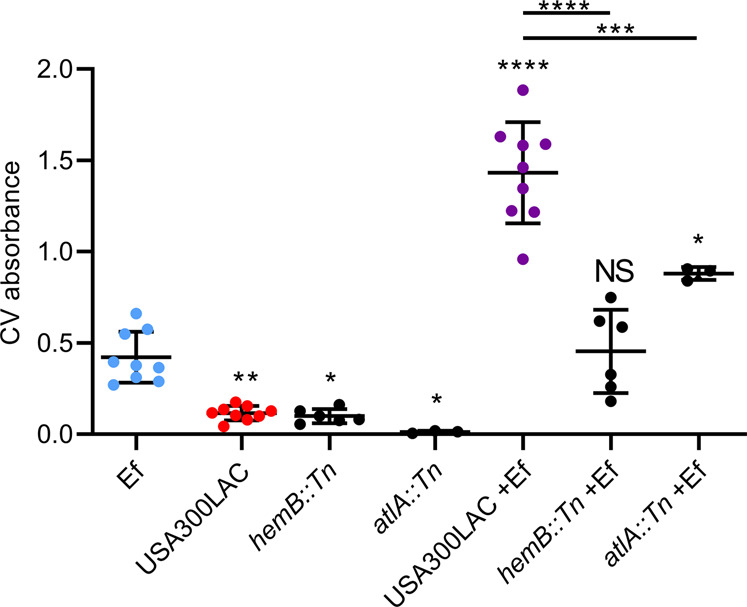
Effect of heme-deficient *S. aureus* mutant on dual-species biofilm augmentation. Parental strain *S. aureus* USA300LAC, the *hemB* transposon mutant and the *atlA* transposon mutant were grown alone or in combination with *E. faecalis* (Ef) for five days before biofilm was quantified with crystal violet (CV). Data shows mean and SD (*N* = 6). *p < 0.05, **p < 0.01, ***p < 0.001 and ****p < 0.0001 when compared to Ef alone, or as otherwise indicated by brackets, using 1-way ANOVA with Tukey’s post-hoc tests.

Consistent with our hypothesis, the *S. aureus hemB* mutant defective in heme biosynthesis did not give rise to augmented dual-species biofilms (Fig. 6). Notably, the single-species parental *S. aureus* biofilm levels were comparable to the *hemB* mutant, suggesting that growth restriction of this mutant in the absence of heme supplementation is unlikely to account for the drastic reduction in dual-species biofilms. However, this does not rule out the possibility that the *hemB* mutant is outcompeted by *E. faecalis* during biofilm formation. Overall, the data indicate that *S. aureus* and *E. faecalis* dual-species biofilm augmentation is dependent on heme biosynthesis in *S. aureus*.

As AtlA is necessary for cell lysis during *S. aureus* biofilm formation [83], we asked whether autolysis contributed to heme release by *S. aureus* to augment dual-species biofilms. When *E. faecalis* and the *S. aureus atlA* transposon mutant were co-cultured, they produced less biofilms than when *E. faecalis* was co-cultured with the *S. aureus* parental strain, but the biomasses were still significantly augmented compared to the *E. faecalis*-only control (Fig. 6). This suggests that AtlA contributes to, but is not exclusively required, for heme release from *S. aureus*.

Recognizing the importance of *S. aureus*-derived heme in driving dual-species biofilm biomass augmentation, we sought to determine if heme and hemoglobin similarly augmented *E. faecalis* biofilm CFU as observed in Fig. 1B. As expected, the un-augmented *cydA::Tn* biofilms had similar CFU to the *E. faecalis* parental strain single species biofilm (Supplementary Fig. 6). Interestingly, while the *E. faecalis* parental strain underwent augmented biomass upon supplementation with hemin or hemoglobin (Fig. 4B) this was not reflected in increased CFU (Supplementary Fig. 6). These data suggest that the mechanism of biofilm augmentation by hemin and hemoglobin may be distinct from that by *S. aureus*, where hemin and hemoglobin elicit increased extracellular matrix production rather than elevating cell numbers. Further work to understand these differences is worthwhile and could inform the induction of *E. faecalis* stress responses to heme/hemoglobin.

### *E. faecalis* Gelatinase E (GelE) is involved in using heme synthesized by *S. aureus*

Having elucidated the role of heme in dual-species biofilm augmentation, we re-examined the *S. aureus* and *E. faecalis* strains and isolates to understand combinations that did not give rise to augmented biofilms (Fig. 2A, B). We assayed intracellular *S. aureus* heme levels in Newman strain and isolate C37 (both failed to augment dual-species biofilms, Fig. 2A) and found them to have comparable levels as USA300LAC (Supplementary Fig. 7). This suggests that heme biosynthesis in *S. aureus*, though important, may not be the dominant factor governing dual-species biofilm augmentation. For instance, strain differences in heme or hemoprotein release from *S. aureus* would affect the efficiency of cross-feeding, whereas production of biofilm or growth inhibitors might curtail *E. faecalis* biofilm formation to begin with.

**Fig. 7:**
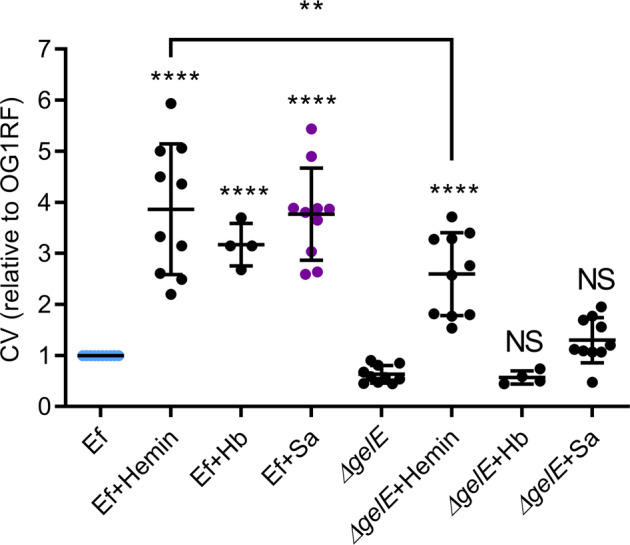
Effects of gelatinase E (*gelE*) deletion on heme-source utilization. Parental strain *E. faecalis* (OG1RF) and respective *gelE* deletion mutant (∆*gelE*), were grown alone or in combination with hemin (25 μg/ml), hemoglobin (Hb, 10 μg/ml) or *S. aureus* (Sa, USA300LAC) for five days before biofilm was quantified with crystal violet (CV), Data shows mean and SD (*N* ≥ 4). ****p < 0.0001 when compared to respective Ef controls using 1-way ANOVA with Bonferroni’s post-hoc tests.

Of the *E. faecalis* strains and isolates, it was notable that OG1X, a sister clone of OG1RF lacking in gelatinase activity [84, 85], was not augmented by USA300LAC (Fig. 2B). We therefore investigated whether *gelE* was responsible for use of USA300LAC-derived heme. Significant biofilm augmentation of *E. faecalis* OG1RF∆*gelE* only occurred during supplementation with hemin, but not *S. aureus* or hemoglobin (Fig. 7). This result demonstrates that free heme (in the form of hemin), but not conjugated heme (in the form of hemoglobin) could be used to augment biofilms in the *gelE* mutant. Moreover, the inability of the *gelE* mutant to augment biofilms in the presence of USA300LAC (Fig. 7) suggests that heme produced by USA300LAC is also in a conjugated hemoprotein form. Additional post-hoc testing also revealed that hemin-augmented biofilms of *E. faecalis* ∆*gelE* were lower than those of WT, likely due to the established role of GelE in *E. faecalis* biofilm [33] that extend beyond its hemoproteolytic activity.

We then selected a subset of *E. faecalis* strains and isolates (20 out of the original 32, and including OG1RF∆*gelE* as control) to assay for biofilm augmentation by hemin and hemoglobin. Hemin supplementation augmented biofilms in 17 of the 21 tested strains/isolates (Supplementary Fig. 8 and Supplementary Table 3). The four that showed little to no augmentation by hemin (HCG5A4, HCG9A2, TTSHW-EF12 and TTSHW-EF30) also did not show augmentation with hemoglobin and *S. aureus*, consistent with the requirement for a functional respiratory chain in *E. faecalis* biofilm augmentation by hemoglobin and *S. aureus*. For TTSHW-EF30, intrinsically high biofilm levels (≥ four-fold that of OG1RF) may have been less amenable to further augmentation. Of the 17 that were augmented by hemin, 10 were also augmented by hemoglobin (Supplementary Fig. 8 and Supplementary Table 3). Of these ten, eight (80%) were gelatinase-positive whereas two (HCG8E9 and TTSHW-EF16) were gelatinase-negative (Supplementary Table 3). In contrast, out of the seven that were augmented by hemin but not by hemoglobin, only V583 was gelatinase-positive. Together these data suggest that gelatinase activity is important for hemoglobin digestion (heme acquisition) but may not be the exclusive enzyme for this activity and may not be sufficient for heme acquisition.

Of the eight gelatinase-positive strains and isolates that were augmented by both hemin and hemoglobin, five were also augmented by *S. aureus* whereas three were not (Supplementary Fig. 8 and Supplementary Table 3). The three that were not augmented by *S. aureus* (TTSHW-EF9, TTSHW-EF18 and TTSHW-EF28) all produced higher levels of biofilms in the unsupplemented *E. faecalis*-only controls (2.6–2.9× more biofilms than OG1RF). This implies that the ability to extract heme from hemoglobin does not necessarily result in dual-species biofilm augmentation. Though there appear to be additional factors that contribute to the response of *E. faecalis* clinical isolates to *S. aureus*, it remains clear that *gelE* facilitates heme acquisition by *E. faecalis* from hemoglobin and *S. aureus* hemoproteins, highlighting a previously unappreciated role for this established virulence factor in *E. faecalis* polymicrobial biofilm formation.

## Discussion

Despite their co-isolation in biofilm associated infections, the only studies characterizing the molecular interaction between *S. aureus* and *E. faecalis* pertain to vancomycin resistance transfer [55–57]. In this study, we show that *S. aureus* and *E. faecalis* synergize to produce more biofilm and demonstrate that this is dependent on the synthesis of heme by *S. aureus* and cross-feeding by *E. faecalis* to exploit this resource for growth in an oxygen-dependent manner.

To appreciate these findings and provide context to the current literature, it is helpful to understand how *E. faecalis* and *S. aureus* interact with other species. *E. faecalis* produces bacteriocin which reduces growth of *C. perfringens* [21] and also hinders the secretion of botulinum neurotoxin of *C. botulinum* [86]. In oropharyngeal candidiasis, *E. faecalis* bacteriocin, EntV, prevents hyphal morphogenesis of *C. albicans* to suppress hyphal-dependent cytotoxicity [87]. Under iron-limiting conditions, *E. faecalis* secretes L-ornithine which promote siderophore synthesis in *E. coli* [25, 88]. Furthermore, *E. faecalis* suppresses host NF-κB-dependent immune activity to promote the virulence of uropathogenic *E. coli* [89], and in early biofilm stages, AI-2 of *E. faecalis* acts as a chemo-attractant and augments *E. coli* biofilms [90]. *E. faecalis* also promotes biofilm matrix production in *P. aeruginosa* [91] through increasing production of exopolysaccharide Pel. In these studies, the effect of *E. faecalis* on other species is highlighted, yet details on how other species influence *E. faecalis* is conspicuously lacking.

The literature on *S. aureus* interactions with other co-colonizing species is more abundant and has been reviewed [92]. In biofilm-associated infections with *Candida albicans*, *S. aureus* exhibits increased resistance to vancomycin [93], increased mucosal adhesion and pathogenicity [94–96], and increased binding to hyphae via hyphal protein Als3p [97]. By contrast, *S. aureus* interactions with other bacteria appear competitive: *Staphylococcus epidermidis* and *Bacillus subtilis* interferes with *S. aureus* quorum sensing by inhibiting the accessory gene regulatory (*agr*) system, resulting in suppression of virulence genes [98–100]. Additionally, *Pseudomonas aeruginosa*, *Streptococcus pneumoniae* and lactic acid bacteria (LAB) produces toxic products such as pyocyanin, LasA and hydrogen peroxide (H_2_O_2_) to inhibit the growth of *S. aureus* [101–104]. LAB also inhibit the colonization of *S. aureus* by competing for host adhesion sites [105]. As countermeasures, *S. aureus* forms small-colony variants to counter *P. aeruginosa* toxins and produces bacteriocins to inhibit LAB [106, 107]. By contrast, *S. aureus* promotes colonization of *Haemophilus influenzae* within the nasal cavity through hemolysis of erythrocytes and the subsequent release of heme and NAD that drives *H. influenza* growth [108, 109]. Notably, *S. aureus* responds to hemoglobin in nasal secretions to increase colonization by dampening *agr* expression [110].

One challenge in microbial ecology is in understanding how strain differences contribute to mixed species interactions. Here we show that most *S. aureus* strains augment *E. faecalis* biofilm formation but that the converse is not true. Of the 16 tested *S. aureus* strains grown with *E. faecalis*, only Newman and C37 did not show any biofilm augmentation. In the case of Newman, supernatants inhibit biofilm formation by other *S. aureus* strains via a proteinase-sensitive, heat-tolerant and soluble protein [111]. It is possible that this same biofilm-inhibitory protein could also interfere with *E. faecalis* biofilm formation giving rise to antagonism such that dual-species biofilm levels were lower than *E. faecalis-*only biofilms. Of 31 *E. faecalis* strains tested, only five were amenable to biofilm augmentation in dual-species biofilms. For several strains, this may be due to their intrinsically lower biofilm producing capacity in the first place. However, the majority appeared to be able to form biofilms yet behaved differently from OG1RF under the conditions tested and restricting the generalizability of dual-species augmentation. This emphasizes the importance of strain differences and raises questions about intrinsic differences in heme uptake and/or aerobic respiration capacity in different *E. faecalis* strains.

The majority of transposon insertion mutants identified as deficient in undergoing biofilm augmentation in the presence of *S. aureus* were in *menA* (1,4-dihydroxy-2-naphthoate isoprenyltransferase, which converts 1,4-dihydroxy-2-naphthoate to demethylmenaquinone) and *cydA* (cytochrome *d* ubiquinol oxidase subunit I), both of which encode proteins involved in oxidative respiration [60]. For respiration to occur in *E. faecalis*, oxygen and heme are required [60, 112], the latter serving as an essential cofactor for cytochrome *bd* (made up of CydA, CydB and three moieties of cofactor heme) and which acts as the terminal demethylquinol (DMKH_2_) oxidase, generating H_2_O from O_2_ and establishing a proton motive force to generate ATP [113]. Since *E. faecalis* is unable to produce porphyrins required to synthesize heme [78], heme must be supplied exogenously. In our experiments, heme was supplied in the form of hemin (free heme) or hemoglobin (protein-conjugated heme). Both hemin and hemoglobin augmented *E. faecalis* biofilms only under oxic conditions, leading to the conclusion that heme augments *E. faecalis* biofilms by enabling aerobic respiration. Clinically, this could translate to dual-species biofilm being more prominent in sites where oxygen is available, such as superficial wounds, and less of a concern in anoxic regions like the bladder [114, 115].

Through a very different mechanism, the shift from anaerobic to aerobic respiration because of interspecies interactions has been described for *Aggregatibacter actinomycetemcomitans*. Though primarily fermentative, the opportunistic oral pathogen *A. actinomycetemcomitans* switched to respiratory metabolism when grown in the presence of *Streptococcus gordonii* in response to enhanced O_2_ bioavailability during coinfection [116]. Although it was not determined how O_2_ levels were augmented, the assumed provision of electron-acceptors (O_2_) from *S. gordonii* (which requires O_2_ to produce H_2_O_2_) resulted in increased *A. actinomycetemcomitans* growth and persistence, giving rise to the term “cross-respiration”. Opposite to this, *S. aureus* has been shown to increase biofilms when grown in anoxic conditions via a SrrAB-dependent programmed cell lysis, whereas the inactivation of the heme production (*hemB*) permitted greater biofilm growth under oxic conditions [83]. Differences between the previous *S. aureus* study and ours could be due to differences in biofilm assay conditions, in particular the duration of incubation (22 hr used previously compared to 5 d used in our study).

Although exogenous heme utilization has been established for both oxidative respiration and catalase (KatA) stability and activity in *E. faecalis* [117, 118], no heme import machinery has been defined. Incidental to our main findings is the discovery that *cydDC* is essential for heme import. Previously shown to be essential for cytochrome *bd* assembly in *E. coli*, the CydDC proteins are ABC transporters that export redox-active thiol compounds such as cysteine and glutathione [119–122]. In *E. coli*, it has also been suggested that CydDC may bind heme to enhance ATP hydrolysis and thiol export [78, 123–125]. Given that no intracellular heme could be detected in *E. faecalis cydD* or *cydC* mutants cultured in heme-supplemented media, we propose that the CydDC heterodimer may also function as a key, if not the sole, heme importer in this species. However, others have suggested that CydDC is not involved in heme import or cytochrome assembly based on everted *E. coli* membrane vesicles involving a *cydD1* point mutant that showed similar patterns of hemin uptake as those in the parental strain and may instead be important for maintenance of a suitable redox environment in the periplasm for conversion to heme *d* [126, 127]. Further work is needed to rule out the possibility that cytochrome *bd* assembly require CydDC, which could also impact heme levels in the membrane, or that other import mechanisms may exist since heme-dependent KatA activity has been described in *cydABDC* deletion mutants [78]. Moreover, we only performed heme quantification on whole-cell lysates, and fractionation experiments could ascertain if heme is internalized into the cytosol or directly incorporated into the cell membrane.

Loss-of-function mutations to the *cydABDC* operon are enriched in *E. faecalis* isolates resistant to killing in the hemoglobin-rich environment of whole blood [128] and this has been attributed to the induction of respiration in parental strain of *E. faecalis* in the presence of heme which increased vulnerability to neutrophil killing [65]. It is therefore possible, in the context of host infection, that only within the immune-shielded environment of biofilms can heme utilization by *E. faecalis* safely take place without concomitant increasing their susceptibility to immune clearance - hence the importance of *E. faecalis* augmenting biofilms in response to heme.

In vitro, it is unclear why *E. faecalis* augmentation by *S. aureus* occurs in biofilms but not planktonic cells. It has been reported by others (and our own studies agree, data not shown) that *E. faecalis* planktonic growth is unaffected by heme supplementation [118]. We speculate that the spatially confined microenvironment of biofilms, or the unique transcriptional profile of biofilm cells, results in sensitivity to heme-based growth augmentation.

Biofilm augmentation of many *E. faecalis* strains and isolates by hemin but not by hemoglobin or heme-producing USA300LAC suggests that an important secondary *E. faecalis* determinant is involved in this mixed species interaction. Comparative genomics led us to identify GelE as required for *E. faecalis* OG1RF biofilm augmentation in the presence of hemoglobin and USA300LAC. Free heme could augment biofilm formation in the *gelE* deletion mutant, but this strain was unable to extract heme from hemoglobin or *S. aureus* hemoproteins. Together with its established role in biofilms, GelE has previously been shown to hydrolyse hemoglobin [129] which would result in heme release and subsequent import, potentially by CydDC, and incorporation into cytochrome *bd* of *E. faecalis* to allow aerobic respiration to augment biofilms. We speculate that *S. aureus* hemoproteins, whether secreted or released during cell death, may likewise need to be hydrolysed for heme release and subsequent uptake by *E. faecalis*.

In summary, we show that augmentation of *S. aureus* and *E. faecalis* dual-species biofilms requires the biosynthesis of heme by *S. aureus* which facilitates oxidative respiration in *E. faecalis*. In most cases, successful cross-feeding of heme (likely in the form of secreted hemoproteins) will require gelatinase-mediated heme acquisition by *E. faecalis*. It is conceivable that the increased energy derived from aerobic respiration enables production of complex and “expensive” extracellular biofilm-associated proteins like EpaOX or surface-anchored pili (EbpABC) [130]. Since *epaOX* (along with *fsrA, gelE* and *epaI*) correlate with biofilm-associated antibiotic resistance [75], the potential impact of this inter-species interaction on antibiotic efficacy is worth considering, especially since this has been demonstrated for other mixed species interactions [131, 132]. The findings of this study highlight the importance of understanding inter-species interactions in biofilms and underscore the usefulness of identifying potentiating determinants, like heme, to develop interventions relevant in a complex host setting.

## Supplementary information

supplemenary figures, tables, materials and methods
