# Supplementary material for: Heme cross-feeding can augment Staphylococcus aureus and Enterococcus faecalis dual species biofilms
Source: ISME J. 2022 May 19;16(8):2015–26. doi: 10.1038/s41396-022-01248-1 (PMC9296619; doi:10.1038/s41396-022-01248-1)
Supplement: Supplementary file 1 — supplemenary figures, tables, materials and methods [file 41396_2022_1248_MOESM1_ESM.docx]

**Supplementary Figure 1**

**a. b.**

**Supplementary Fig 1.** *E. faecalis* glycosyltransferase *epaOX* mutants or wild-type OG1RF were grown alone or with *S. aureus* (Sa) for 5 days before biofilm quantification with crystal violet. Results are plotted as **a** fold-change of CV stain relative to the respective non-supplemented single-species strain controls, with mean and SD displayed and **b** relative to OG1RF control and indicated the mean and SD values of CV absorbance. **p*<0.05*, **p*<0.01, ****p*<0.001 and ****p<0.0001 by Dunnett's post-hoc tests against first dataset of the respective groups (N=6).

**Supplementary Figure 2**

**Supplementary Fig 2.** Oxygen consumption rate (OCR) of *E. faecalis* biofilms. *E. faecalis* biofilms grown for 5 days in TSB with and without hemin (25 µg/ml) and hemoglobin (10 µg/ml) supplementation in 96-well format Seahorse cell culture plates. Planktonic cells were washed off and the biofilm resuspended in PBS for three baseline measurements. After which TSB was injected (arrow) and additional readings were taken up to 1 hr. Data shows mean and SD of OCR (N=3).

**Supplementary Figure 3**

**Supplementary Fig 3.** Biofilm kinetics of *E. faecalis* biofilms. *E. faecalis* biofilms grown in 96-well format in the presence of *S. aureus* (Sa) (1:1) and hemin (25 µg/ml) over 5 days were quantified by crystal violet (CV) staining. Data shows mean and SD of absorbance. **p*<0.05, ****p*<0.001 and *****p*<0.0001 when compared to *E. faecalis*-only biofilms of the same time point, or between *E. faecalis* augmented with *S. aureus* or hemin supplementation using Tukey’s post-hoc test (N≥3).

**Supplementary Figure 4**

**Supplementary Fig 4.** Planktonic *E. faecalis* cells cultured alone, or with *S. aureus* (1:1), for 1 day and assayed for CFUs by plating on selective agar. Data shows mean and SD. **p*<0.05, using Dunnet’s post-hoc test against the respective OG1RF control (N=3).

**Supplementary Figure 5**

**a. b.**

**c.**

**Supplementary Fig 5.** Growth kinetics of *S. aureus hem* mutants and relative heme values. Growth of *S. aureus* wild-type USA300LAC (WT) and *hem* transposon mutants in **a** TSB or **b** TSB supplemented with 2.5 µg/ml hemin. Data shows mean and SD (N≥3). **c** Cell pellets of WT and respective *hem* mutants were collected, lysed and intracellular heme levels were quantified by LC-MS. Results show mean values and SD (N=3) with *****p*<0.0001 when compared to WT using 1-way ANOVA with Dunnet’s post-hoc test.

**Supplementary Figure 6:**

**Supplementary Fig. 6.** Biofilm *E. faecalis* CFU counts. *E. faecalis* biofilms of wildtype OG1RF and the *cydA* transposon mutant were grown for 5 days with and without co-culture with *S. aureus* (Sa, 1:1), supplementation of hemin (25 µg/ml) or hemoglobin (Hb, 10 µg/ml). Biofilms were collected and CFU/well determined using selective agar. Data shows mean and SD (N=3) with **p<0.01 compared to the OG1RF control, using 1-way ANOVA and Tukey’s post-hoc tests.

**Supplementary Figure 7**

**Supplementary Fig 7.** *S. aureus* heme quantification. Cell pellets of USA300LAC, Newman and clinical isolate C37 were collected, lysed and intracellular heme quantified by LC-MS. Results show mean values and SD (N=3) with no significance detected by 1-way ANOVA.

**Supplementary Figure 8**

**Supplementary Fig 8.** Augmentation of biofilms in *E. faecalis* laboratory strains and clinical isolates by hemin, hemoglobin and *S. aureus*. Five laboratory strains/mutants of *E. faecalis* (OG1RF, OG1RFΔ*gelE*, OG1X, JH2.2 and V583), together with 16 *E. faecalis* patient isolates, were grown for 5 days alone, with hemin (5 μg/ml), with hemoglobin (Hb, 10 μg/ml) or with *S. aureus* (Sa, USA300LAC) before biofilm was quantified by CV. Results show biofilm levels normalized to OG1RF-only controls, with mean and SD (N≥3). Points are colored (red, green or blue) if significantly different to respective Ef-only biofilms by *p*<0.01 using 1-way ANOVA with Dunnet’s posthoc test (details in Supplementary Table 3). Strains/isolates in bold are gelatinase positive.

**Supplementary Table 1**

Detailed statistics for Figure 2a.

| *S. aureus* strain/isolate | Sa-alone vs Sa+OG1RF | |
| --- | --- | --- |
|  | p-value | Fold-change |
| USA300 | **** | 19.94 |
| Newman | ns | 2.73 |
| HG001 | **** | 1.83 |
| HG003 | **** | 2.38 |
| MN8 | **** | 4.12 |
| ISP479 | **** | 2.42 |
| C01 | **** | 8.51 |
| C04 | **** | 19.87 |
| C07 | **** | 3.62 |
| C10 | **** | 5.08 |
| C14 | **** | 21.69 |
| C21 | **** | 5.43 |
| C32 | **** | 7.61 |
| C37 | ns | 1.37 |
| C41 | **** | 8.26 |
| C50 | **** | 9.54 |

**Supplementary Table 2**

Detailed statistics for Figure 2b.

| *E. faecalis* strain/isolate | Ef-only biofilm levels (relative to OG1RF) | Ef-only  biofilm levels | Ef-alone vs Ef+USA300 | |
| --- | --- | --- | --- | --- |
|  |  |  | p-value | Fold-change |
| OG1RF | 1.000 |  | **** | 3.10 |
| OG1X | 0.339 | low (<0.5 of OG1RF) | ns | 1.04 |
| JH2.2 | 0.119 | low (<0.5 of OG1RF) | ns | 4.43 |
| V583 | 1.110 | similar to OG1RF | ns | 0.64 |
| VRE122 | 0.360 | low (<0.5 of OG1RF) | ns | 0.25 |
| VRE222 | 0.486 | low (<0.5 of OG1RF) | ns | 0.90 |
| VRE322 | 0.403 | low (<0.5 of OG1RF) | ns | 2.41 |
| VRE422 | 0.285 | low (<0.5 of OG1RF) | ns | 0.65 |
| VRE522 | 0.207 | low (<0.5 of OG1RF) | ns | 1.03 |
| UTIEF2340 | 0.191 | low (<0.5 of OG1RF) | ns | 1.22 |
| UTIEF5245 | 0.177 | low (<0.5 of OG1RF) | ns | 4.15 |
| UTIEF2116 | 0.261 | low (<0.5 of OG1RF) | ns | 1.52 |
| HCG5A4 | 0.660 | similar to OG1RF | ns | 0.98 |
| HCG5H8 | 0.223 | low (<0.5 of OG1RF) | ns | 0.57 |
| HCG9A2 | 0.483 | low (<0.5 of OG1RF) | ns | 1.44 |
| HCG2E2 | 1.044 | similar to OG1RF | ns | 0.66 |
| HCG8D2 | 0.254 | low (<0.5 of OG1RF) | ns | 2.06 |
| HCG2C3 | 0.396 | low (<0.5 of OG1RF) | ns | 1.57 |
| HCG8E9 | 0.307 | low (<0.5 of OG1RF) | ns | 2.48 |
| TTSHW-EF05 | 1.578 | similar to OG1RF | **** | 2.06 |
| TTSHW-EF09 | 2.661 | high (>2 of OG1RF) | ns | 0.89 |
| TTSHW-EF12 | 1.537 | similar to OG1RF | ns | 1.69 |
| TTSHW-EF13 | 1.073 | similar to OG1RF | **** | 3.04 |
| TTSHW-EF15 | 0.221 | low (<0.5 of OG1RF) | ns | 0.17 |
| TTSHW-EF16 | 0.750 | similar to OG1RF | **** | 3.68 |
| TTSHW-EF18 | 2.928 | high (>2 of OG1RF) | ns | 1.23 |
| TTSHW-EF24 | 1.542 | similar to OG1RF | **** | 2.15 |
| TTSHW-EF28 | 2.585 | high (>2 of OG1RF) | ns | 1.35 |
| TTSHW-EF30 | 4.404 | high (>2 of OG1RF) | ns | 1.15 |
| TTSHW-EF36 | 2.058 | high (>2 of OG1RF) | ns | 0.62 |
| TTSHW-EF42 | 0.232 | low (<0.5 of OG1RF) | ns | 1.14 |
| TTSHW-EF43 | 1.140 | similar to OG1RF | **** | 3.01 |

**Supplementary Table 3**

Detailed statistics for Supplementary Figure 5.

|  | **Augmentation by Hemin** | | **Augmentation by Hemoglobin** | | **Gelatinase activity** | **Augmentation by *S. aureus*** | | **Ef-only biofilm levels** | |
| --- | --- | --- | --- | --- | --- | --- | --- | --- | --- |
| **Strain** | **Fold-change** | **Significance (*p*<0.01)** | **Fold-change** | **Significance (*p*<0.01)** | **+/-** | **Fold-change** | **Significance (*p*<0.01)** | **Relative to OG1RF** | **Levels  (ok if >0.5 & <2)** |
| OG1RF | **3.26** | ******** | **3.76** | ******** | **Positive** | **3.10** | ******** | 1.00 | ok |
| TTSHW-EF05 | **2.36** | ******* | **2.45** | ******* | **Positive** | **2.06** | ****** | 1.58 | ok |
| TTSHW-EF13 | **3.30** | ******** | **3.88** | ******** | **Positive** | **3.04** | ******** | 1.07 | ok |
| TTSHW-EF24 | **2.43** | ******** | **2.61** | ******** | **Positive** | **2.15** | ******* | 1.54 | ok |
| TTSHW-EF43 | **3.00** | ******** | **3.44** | ******** | **Positive** | **3.01** | ******** | 1.14 | ok |
| TTSHW-EF16 | **5.17** | ******** | **6.30** | ******** | Negative | **3.68** | ****** | 0.75 | ok |
| TTSHW-EF09 | **1.99** | ******** | **1.95** | ******** | **Positive** | 0.89 | NS | 2.66 | **High** |
| TTSHW-EF18 | **2.41** | ******** | **2.33** | ******* | **Positive** | 1.23 | NS | 2.93 | **High** |
| TTSHW-EF28 | **2.44** | ******** | **2.34** | ******** | **Positive** | 1.35 | NS | 2.59 | **High** |
| HCG8E9 | **4.05** | ******** | **3.21** | ****** | Negative | 2.48 | NS | 0.31 | Slightly low |
| V583 | **2.15** | ******** | 0.99 | NS | **Positive** | 0.64 | NS | 1.11 | ok |
| OG1X | **4.11** | ******** | 1.15 | NS | Negative | 1.04 | NS | 0.34 | Slightly low |
| OG1RFΔgelE | **4.46** | ******** | 1.12 | NS | Negative | 2.00 | NS | 0.39 | Slightly low |
| HCG2C3 | **4.21** | ******* | 2.98 | NS | Negative | 1.57 | NS | 0.4 | Slightly low |
| HCG8D2 | **5.89** | ****** | 3.38 | NS | Negative | 4.43 | NS | 0.25 | Low |
| JH2.2 | **16.15** | ******* | 8.46 | NS | Negative | 4.14 | NS | 0.12 | Low |
| UTIEF5245 | **3.51** | ******** | 2.96 | NS | Negative | 2.06 | NS | 0.18 | Low |
| TTSHW-EF30 | 1.40 | NS | 1.44 | NS | **Positive** | 1.15 | NS | 4.4 | **Very High** |
| HCG5A4 | 1.40 | NS | 1.18 | NS | Positive (little) | 0.98 | NS | 0.66 | ok |
| HCG9A2 | 2.13 | NS | 1.42 | NS | Positive (little) | 1.44 | NS | 0.48 | Slightly low |
| TTSHW-EF12 | 2.69 | NS | 2.92 | NS | Negative | 1.69 | NS | 1.54 | ok |

**Supplementary Materials and Methods**

**Bacterial Strains and Growth Conditions**

The *Enterococcus faecalis* laboratory strains of OG1RF (1), OG1X (2, 3) and V583 (4) are well described, OG1RFΔ*epaOX* and complement were from Stéphane Mesnage (5) and OG1RFΔ*gelE* was from Lynn Hancock (6). The *Staphylococcus aureus* USA300 strain used is an erythromycin-sensitive LAC (CA-MRSA USA300-0114, AH1263) (7) provided by Alexander R. Horswill, as was HG001 and HG003. ISP479 and MN8 were from Yang Liang and Newman was provided by Eric P. Skaar (8). Clinical isolates of *E. faecalis* (TTSHW-EF05 to EF43) and *S. aureus* isolates (C01-C50) are from Tan Tock Seng Hospital, Singapore. Note that TTSHW-EF43 was labelled as TTSHW-EFS01 in a previous publication (9). Other *E. faecalis* isolates were from Scott J. Hultgren (UTI isolates UTIEF2116, UTIEF2340 and UTIEF5245), Barnes-Jewish Hospital microbiology laboratory in St. Louis, MO (VRE blood isolates 122 to 422), Phillip I. Tarr (healthy children’s gut isolates, HCG8D2 to HCG2E2) (10).

**Molecular cloning**

The primers used in this study are listed in Supplementary Data. Transformants were screened in respective selection agar as follows: (A) *E. coli* strains, LB with 500 µg/mL erythromycin (pGCP213); and (B) *E. faecalis* strains, BHI with 25 µg/mL erythromycin (pGCP213). Construction of *E. faecalis* knock-out and complement mutants were done by allelic replacement via a temperature-sensitive shuttle vector described previously (11). For the construction of ∆*cydA*, ∆*cydB*, ∆*cydC* and ∆*cydD*, pGCP213 vector was linearize using NotI and BamHI restriction enzymes (New England Biolabs, USA). For the construction of ∆*cydB+cydB* and ∆*cydD+cydD* complement strains, pGCP213 vector with *srtA* promoter (pGCP213::P*_srtA_*) was linearize using iPCR_pGCP213::PsrtA_F and iPCR_pGCP213::PsrtA_R primers. Linearized vectors and respective inserts were ligated using In-Fusion® HD Cloning Kit (Clontech, Takara, Japan) and transformed into Stellar^TM^ competent cells. Successful plasmid constructs were verified through Sanger sequencing and subsequently extracted and transformed into OG1RF. Transformants were selected with erythromycin at 30 °C, then passaged at non-permissive temperature at 42 °C with erythromycin to select for bacteria with successful plasmid integration into the chromosome. For plasmid excision, bacteria was serially passaged at 30 °C without erythromycin for erythromycin-sensitive colonies. These erythromycin-sensitive colonies were then subjected to PCR screening using respective screening primers for detection of deletion mutant or chromosomal complementation of *cydA*, *cydB*, *cydC* and *cydD*.

**Biofilm Assay**

Planktonic cells were removed by washing wells thrice with 250 μl PBS, fixed with methanol (Merck, Singapore) for 1 min and stained for 5-10 min in 0.1% Crystal Violet (Sigma Aldrich, United States). Excess stain was washed away with three additional washes of 250 µl water. The plates were dried, then eluted with 30% acetic acid (Merck, Singapore) for 1 h at RT. Absorbance was measured at 595 nm with Tecan Infinite M200 PRO Spectrophotometer (Tecan, Switzerland).

For CFU determination, 100 µl of planktonic cells from two replicate wells were collected by gentle pipetting. The collected planktonic cells were pooled and mixed with 800 µl of PBS. Followingly this, remaining planktonic cells were removed by washing thrice with 250 µl PBS. An additional 250 µl PBS was added and a sterile 10 µl tip was used to thoroughly scrape along the sides and base of the well. Scraped biofilms from four replicate wells were thoroughly resuspended by vigorous pipetting to break apart biofilms before being pooled. 10-fold serial dilutions were performed and spots of 5 µl and 4 µl were made in replicates on BHI+rifampicin and MRSA Select II plates respectively. Plates were incubated overnight at 37 ^o^C and CFUs determined the next day.

**Transposon Library Screen**

The *E. faecalis* transposon library generated with OG1RF mariner transposon sequence consisted of 14 978 individual mutants cryogenically stored in a 96-well format (12). Cryoreplicators were used to inoculate overnight cultures of the library cryostock (13) to a new 96-well plate with 200 µl TSB and the plates were incubated overnight at 37 ^o^C.

**UPLC-MS for heme quantification from cell pellet**

Heme quantification was performed according to previous publication (14) with some modification. Briefly, proteins were precipitated by adding 1 mL of acetonitrile to the sample before 10 μL of concentrated (35-37%) HCl was added. Samples were incubated for 20 min at RT and vortexed for 5 min. To create a two-phase liquid-liquid system, saturated MgSO4, and 0.1 g NaCl was added. The samples were then centrifuged, and the organic phase was collected and injected into a BEH C18 (2.1x100 mm;1.7 µm) column. The mobile phase A consisting of 0.1% formic acid and mobile phase B consisting of acetonitrile with 0.1% formic acid was used. Xevo TQ-S mass spectrometer (Waters) was run in ESI positive (MRM) mode with 2.5 kV capillary voltage for screen time of up to 7 min. Three replicate readings were averaged to obtain the final value of each biological replicate, and three biological replicates were quantified for each strain.

**Supplementary References**

1. Keogh D, Tay WH, Ho YY, Dale JL, Chen S, Umashankar S, et al. Enterococcal metabolite cues facilitate interspecies niche modulation and polymicrobial infection. Cell Host Microbe. 2016;20(4):493-503.

2. Ike Y, Craig RA, White BA, Yagi Y, Clewell DB. Modification of *Streptococcus faecalis* sex pheromones after acquisition of plasmid DNA. Proc Natl Acad Sci U S A. 1983;80(17):5369-73.

3. Su YA, Sulavik MC, He P, Makinen KK, Makinen PL, Fiedler S, et al. Nucleotide sequence of the gelatinase gene (*gelE*) from *Enterococcus faecalis subsp. liquefaciens*. Infect Immun. 1991;59(1):415-20.

4. Nielsen HV, Flores-Mireles AL, Kau AL, Kline KA, Pinkner JS, Neiers F, et al. Pilin and sortase residues critical for endocarditis- and biofilm-associated pilus biogenesis in *Enterococcus faecalis*. J Bacteriol. 2013;195(19):4484-95.

5. Smith RE, Salamaga B, Szkuta P, Hajdamowicz N, Prajsnar TK, Bulmer GS, et al. Decoration of the enterococcal polysaccharide antigen EPA is essential for virulence, cell surface charge and interaction with effectors of the innate immune system. PLoS Pathog. 2019;15(5):e1007730.

6. Thomas VC, Hiromasa Y, Harms N, Thurlow L, Tomich J, Hancock LE. A fratricidal mechanism is responsible for eDNA release and contributes to biofilm development of *Enterococcus faecalis*. Mol Microbiol. 2009;72(4):1022-36.

7. Boles BR, Thoendel M, Roth AJ, Horswill AR. Identification of genes involved in polysaccharide-independent *Staphylococcus aureus* biofilm formation. PLoS One. 2010;5(4):e10146.

8. Hammer ND, Skaar EP. Molecular mechanisms of *Staphylococcus aureus* iron acquisition. Annu Rev Microbiol. 2011;65:129-47.

9. Chong KKL, Tay WH, Janela B, Yong AMH, Liew TH, Madden L, et al. *Enterococcus faecalis* modulates immune activation and slows healing during wound infection. J Infect Dis. 2017;216(12):1644-54.

10. Denno DM, Shaikh N, Stapp JR, Qin X, Hutter CM, Hoffman V, et al. Diarrhea etiology in a pediatric emergency department: a case control study. Clin Infect Dis. 2012;55(7):897-904.

11. Nielsen HV, Guiton PS, Kline KA, Port GC, Pinkner JS, Neiers F, et al. The metal ion-dependent adhesion site motif of the *Enterococcus faecalis* EbpA pilin mediates pilus function in catheter-associated urinary tract infection. mBio. 2012;3(4):e00177-12.

12. Kristich CJ, Nguyen VT, Le T, Barnes AM, Grindle S, Dunny GM. Development and use of an efficient system for random mariner transposon mutagenesis to identify novel genetic determinants of biofilm formation in the core *Enterococcus faecalis* genome. Appl Environ Microbiol. 2008;74(11):3377-86.

13. Keogh D, Lam LN, Doyle LE, Matysik A, Pavagadhi S, Umashankar S, et al. Extracellular electron transfer powers *Enterococcus faecalis* biofilm metabolism. mBio. 2018;9(2).

14. Fyrestam J, Ostman C. Determination of heme in microorganisms using HPLC-MS/MS and cobalt(III) protoporphyrin IX inhibition of heme acquisition in *Escherichia coli*. Anal Bioanal Chem. 2017;409(30):6999-7010.
